# Estimating the consequences of a COVID-19 super spreader: A stochastic model of a night on the town

**DOI:** 10.1177/14034948211031400

**Published:** 2021-07-25

**Authors:** Nils Henrik Kolnes, Snorre Nilsen Eikeland, Tor Albert Ersdal, Geir Sverre Braut

**Affiliations:** 1 Analytic department, Helse Stavanger HF, Norway; 2 Chief Financial Officer, Helse Stavanger HF, Norway; 3 Research department, Helse Stavanger HF, Norway

**Keywords:** COVID-19, Super spreader event, modelling

## Abstract

A stochastic model estimated the consequences of a COVID-19 super spreader event occurring in the local municipality of Stavanger, Norway as a result of a night on the town. The model imposed different infection control regulations and compared these different scenarios. For Stavanger’s 161 locations of service, secondary transmissions from a super spreader event was estimated to infect a median of 37, requiring the quarantining of 200 guests given no infection control regulations, 23 and 167 when imposing social distancing regulations and other hygienic infection control measures, 7 infected and 63 quarantined guests with restrictions placed on the guest capacity, and 4 infected and 57 quarantined guests with both forms of restriction in use.

## Introduction

COVID-19 spreads with a high individual-level variation in secondary attack rate. A study by Endo et al. estimates that the individual variation in transmission of COVID-19 follows a negative binomial distribution, with a dispersion factor of 0.1 (0.05–0.2) 95% confidence interval (CI) [1], meaning that 80% of the spread is caused by 10% of the people infected [2]. This skewness in spread gives rise to the possibility of super spreader events, where one person can infect a large group of people given the right circumstances.

Norway quickly gained control over the COVID-19 pandemic during the spring of 2020. The pandemic was all but eradicated by the end of April 2020 and the country started reopening. At first, all restaurants and bars were restricted to a maximum of 50 guests [3]. On 15 June this restriction was changed to 200 guests. However, by the end of summer that year, local outbreaks had again started to occur within several municipalities throughout Norway. The source of these outbreaks were events where people had gathered in mass [4], such as at house parties and in bars and restaurants. One of the major outbreaks originated from three bars during one night, and resulted in the local government re-enforcing severe restrictions on the local community [5].

On 1 June, 2020 a change to the national legislation was made, giving local government the power to remove the licence to serve alcohol of any restaurant or bar if a violation to the infection control measures was made.

This paper is intended to be a management tool for the evaluation of local and national infection control regulations that are applied to the nightlife industry with regard to the impacts of potential super spreader events. Models that allow for local adaptation are useful given variation in how COVID-19 can affect different geographical areas [6].

The objective of the model presented in this paper is to simulate the consequences of a super spreader event originating from an infected case (or an infected cohort) going for a night on the town. The assumption made for the analysis was that the event had occurred. The simulation estimated the consequences of such an event. The analysis also aimed to compare different scenarios based on the previous and current restrictions imposed on restaurants and bars. The effects of social distancing and other infection control measures that the restaurants and bars undertook prior to the pandemic were also considered.

As a final analysis, the effect of shutting down restaurants and bars that infringed measures was simulated and compared across scenarios.

## Methods

### The model

A stochastic model was built in RStudio [7] to simulate the potential consequence of a super spreader event occurring in Stavanger, Norway. The base for the model was a COVID-19 infected case/s visiting bars, restaurants and nightclubs (designated i in this article) during an evening/night in Stavanger.

Data containing the guest capacity for each restaurant and bar in Stavanger and the surroundings were supplied by the local government. There was a total of 161 restaurants, bars and nightclubs $\left(N_{l}\right)$ . To simplify further calculations, the guest capacity $\left(\Gamma_{i}\right)$ was divided into two categories: maximum = 50 (91 locations) and maximum = 200 (70 locations).

The analysis relied on inputs from local data and knowledge of social conditions; the source code used for this model can be accessed at Github [8].

### The scenarios

The analysis simulated four different possible government strategies (j) towards maintaining control over the COVID-19 pandemic (see Table I). Each scenario either imposed a restricting guest capacity, other infection control measures, both or neither. The ‘other infection control measures’ included several hygienic and social distancing measures and was evaluated in the model as a factor (σ) that was set to be normally distributed, N(0.8,0.2) , for Scenarios 2 and 4, and 1 for Scenarios 1 and 3.

**Table I.** The different scenarios j∈{1,2,3,4} simulated in this study. Scenario 1 represents the local nightlife without restrictions; Scenario 2 represents a situation with imposed infection control measures on bars and restaurants, but no restriction on guest capacity; Scenarios 3 and 4 are versions of Scenarios 1 and 2 but with a restriction on guest capacity.

Scenario (j)	Max guest capacity $\left(\Gamma_{j}\right)$	Infection control factor $\left(\sigma_{j}\right)$
1	200	1
2	200	N(0.8,0.2)
3	50	1
4	50	N(0.8,0.2)

Scenario 1 represented the local nightlife before the pandemic and governmental regulations; Scenario 2 simulated today’s situation with regard to guest capacity and infection control measures; Scenarios 3 and 4 were different variations of the situation during the reopening phase but before allowing a maximum of 200 guests.

### The simulation

Before starting the simulation, the model was initiated by filling up the bars and restaurants with guests. Each location sampled the occupancy rate $\left(\rho_{i}\right)$ from a beta distribution ( α=5,β=3 ) to infer a skewed distribution of guests at different locations, representing the fact that some locations are more popular than others. The occupancy rate for each location was then multiplied by the given location’s guest capacity $\left(\Gamma_{i}\right)$ , the infection control factor $\left(\sigma_{i}\right)$ , and rounded to nearest number to get the total number of susceptible guests ( $s_{i}$ ) for each location at each scenario,

(1) $$s_{i}=\sum_{i=1}^{N_{l}}\rho_{i}\Gamma_{i}\sigma_{j},$$

giving the total number of susceptible people being out for a night on the town,

(2) $$s_{i}=\sum_{i=1}^{N_{l}}s_{i}.$$

After populating the model, simulation of the super spreader was initiated. For simplification purposes, the guests at each location were stationary during the whole simulation and only the super spreader moved between locations. For each simulation the super spreader could visit K locations where K∈{1,2,3} . Each location visited was indexed k and sampled from Nl with a probability vector being each location’s occupancy rate $\left(\rho_{i}\right)$ to simulate that it is more likely for a random person/cohort to visit popular places. For each location the super spreader visited, a fraction of the guests ( $\epsilon_{k}$ ) were infected. To estimate a value for $\epsilon_{k}$ , a constant secondary attack rate, *SAR*, was given and multiplied by the occupancy rate to reflect the assumption that the risk of being infected reduced the more unoccupied space there was available to each guest,

(3) $$\epsilon_{k}=SAR\cdot \rho_{k}.$$

The SAR value was determined to be constant at 0.25 in this analysis to simulate that the super spreader infected 25% of the guests at locations that had 100% occupancy rate.

Finally, to evaluate the number of people that were exposed at each location, $e_{k}$ ,

(4) $$e_{k}=\epsilon_{k}s_{k},$$

and the total number of exposed people for each simulation, E ,

(5) $$E=\sum_{k=1}^{k}e_{k}.$$

The number of quarantined susceptible guests, Sq , were estimated assuming that all people that visited the same location as the infected would be traced and put in quarantine for 10 days,

(6) $$s_{q}=\sum_{k=1}^{k}s_{k}.$$

### Simulating closing down bars and restaurants violating regulations

The final analysis followed the method described above with one addition. After setting each location’s guest capacity $\left(\Gamma_{i}\right)$ multiplied by the infection control measure $\left(\sigma_{i}\right)$ , the model checked that the maximum occupancy rate was less than 90%. This was only done for Scenarios 2 and 4 (named Scenarios 2.1 and 4.1), where the maximum occupancy rate was assumed to be 80%, and infection control measures were assumed to have been imposed by the local government. Locations with occupancy rates greater than or equal to 90% were closed and the number of guests was set to 0. An occupancy rate of 90% was chosen (instead of 80%) to account for possible local variation.

This study is part of the ongoing predictions performed at the analytic department for emergency preparedness efforts of Stavanger University Hospital. The legal basis for this study was the regulations pertaining to leadership and quality improvement in the health and care services in Norway [9]. No use was made of information relating to single individuals. There was therefore no need for any particular legal or ethical approval as personal information was not used for this study.

## Results

Table II presents the median and the 90% quantile number of exposed guests, *E*, susceptible guests in quarantine, *Sq*, and the total number of susceptible guests, *S*, presented for all scenarios.

**Table II.** The estimated median and 90% quantile for number of exposed guests, *E*, and susceptible quarantined guests, *Sq*, as well as the estimated total number of susceptible guests, *S*, for each scenario.

Scenario (*j*)	Median (E)	90% quantile (*E*)	Median (*Sq*)	90% quantile (*Sq*)	Total (*S*)
1	37	70	200	365	11,786
2	23	48	167	313	9329
2.1	22	46	146	276	6267
3	7	14	63	104	5045
4	4	9	57	96	4495
4.1	4	8	56	96	3675

**Figure 1.**
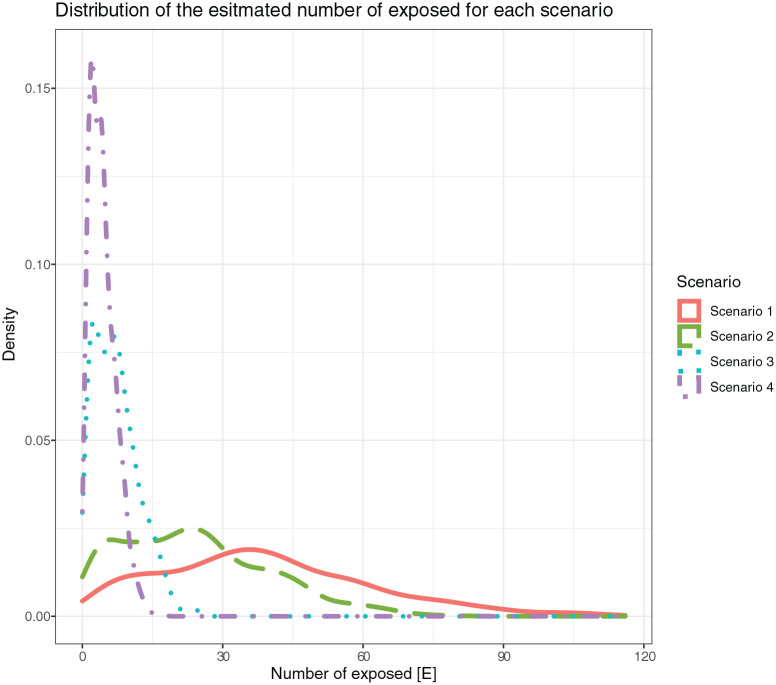
Distribution of estimated number of exposed guests *E* for Scenarios 1 through 4.

The resulting distributions of the number of exposed guests, *E*, for each scenario is presented in Figure 1. It is clear that the number of exposed guests, *E*, for Scenario 2 was approximately two-thirds of *E* cases in Scenario 1. Similarly, the number of *E* in Scenarios 3 and 4 were one-fifth and one-ninth the exposed cases in Scenario 1, respectively, showing the effectiveness of the different simulated enforced regulations.

**Figure 2.**
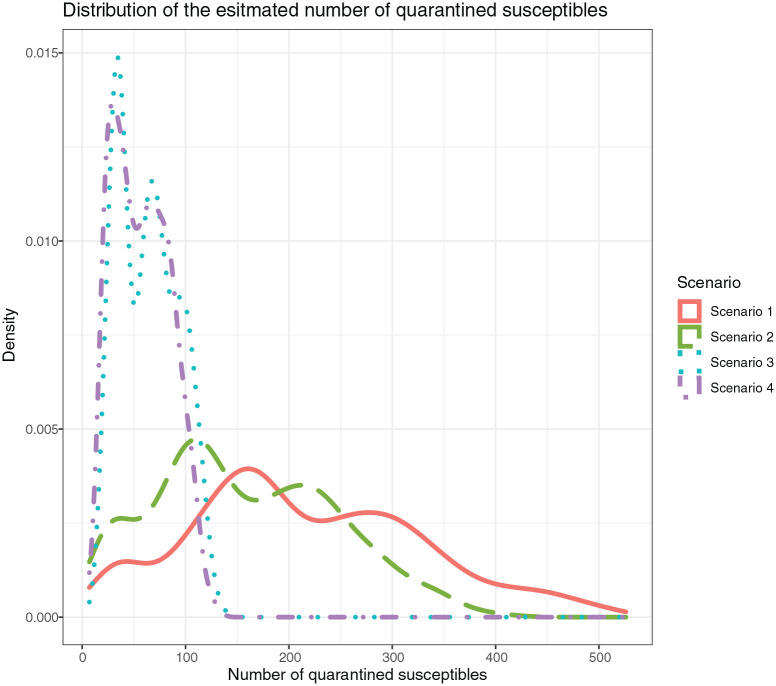
Distribution of estimated number of quarantined susceptible guests, *Sq*, for Scenarios 1 through 4.

Looking at the distributions for the quarantined susceptible guests, *Sq*, in Figure 2, Scenarios 3 and 4 followed a similar trend and defined similar consequence regions. The same could be observed for Scenarios 1 and 2. This indicated that the infection control measures, σ , had little effect on the number of *Sq*. The estimated number of *Sq* clearly indicated the insignificant effect of the intervention (Table II).

The total number of susceptible guests, *S*, for each scenario followed an expected trend, since the maximum guest capacity and the infection control factor controlled the amount of guests that could be out at the same time.

Comparing the results for Scenarios 2.1 and 4.1 with Scenarios 2 and 4 respectively, the total number of estimated susceptible guests reduced by a third. A small reduction was observed for the exposed guests, whereas a larger, yet still relatively small reduction was seen in the quarantined susceptible guests.

## Discussion

The results obtained from this study could be used as the model for a local community health development. It is important to note that the model had several simplifications and assumptions made in relation to input parameters. For instance, the *SAR* value was set with [1] and [10] as a basis and the infection control factor, σ , was based on [11], as well as given a level of uncertainty, to simulate local differences for each location’s capacity and willingness to adapt to the infection control measures imposed on them, however, neither parameter was statistically derived from data. A third parameter that played a major role with regard to the values in the estimated results was the beta distribution that was set to make some restaurants and bars more popular and attractive than others. Different α - and β values, or different distributions, could be set to simulate varying dynamics with respect to which bars and restaurants people visit.

As stated, the model assumed a super spreader event had occurred. This paper, however, says nothing about the probability of such an event occurring, other than the indirect estimate of the number of susceptible guests, *S*, that is, the more people that are out for a night on the town, the higher the probability that one or more will be infectious. However, the probability also depends on other factors, such as the local level of infection and societal behaviour. For future work, a model that estimates the probability of such an event occurring based on the inputs mentioned, as well as other relevant parameters, would be of great value to local and national governments.

The findings from this study could be used to stimulate discussions about the potential outcomes of the different scenarios tested in the model. As an example, the Norwegian government had hoped to allow 500 guests at public functions from 1 September, 2020 [4]. How might this public policy impact on the possible outcomes, given a super spreader event?

Estimated results were also presented to emphasise how the pandemic affects different regions of society. For example, the number of exposed guests is significant from a hospital planning and management perspective and could be used to further simulate a pandemic spread using a SEIR (susceptible, exposed, infected, recovered) model, whereas the number of quarantined susceptible guests is an important consideration from a socioeconomic perspective.

The results presented in Table II illustrate a comparatively large spread in values across the different scenarios. The difference in the relative potential risk of a further spread was also an important factor to consider. The model estimated the consequences based on the *SAR*, but did not account for the *FAR*. Estimating *FAR* values is extremely difficult as the amount of possible scenarios are limitless and the range of outcomes is massive. At one end of the spectrum, the super spreader could be tested the day after the event, giving local government an early warning and allowing them time to put out the fire before it spreads too far. At the other end of the spectrum, the super spreader might not take a test. The exposed guests, *E*, could then potentially spread the virus throughout their social networks and, in the worst case, start a second wave [12]. Looking at such scenarios could be essential to further explain and demonstrate the possible long-term consequences of a super spreader event. Future work could therefore simulate similar events using individual-based models, where outputs would valid for further SEIR modelling of the spread.

The small effect on the number of exposed, *E*, and quarantined susceptible guests, *Sq*, seen by comparing Scenarios 2 and 4 with 2.1 and 4.1 was expected given the precondition set for the analysis: that the super spreader event was happening no matter what conditions were imposed. Fewer open bars and restaurants resulted in the super spreader visiting the other open bars and restaurants that had slightly fewer guests. The more interesting result lay in the number of susceptible guests, *S*. For this parameter, a drop when closing down bars and restaurants was very reasonable, as there were fewer available places to visit. An indirect consequence of this was that the probability of a super spreader event occurring during a night on the town reduced. This could be viewed as a positive outcome for any government.

The question that must be raised, however, is whether the super spreader event happens somewhere else when bars and restaurants close. Will the reduced number of susceptible guests, *S*, go to privately arranged house parties instead? An argument could be made that it is more responsible to allow guests in bars and restaurants, where infection control is regulated and controlled by local authorities. Hence, a *SAR* value would be expected to be higher at privately arranged house parties. Conversely, the number of susceptible guests, *S*, would be expected to be lower at each locally arranged party. Guests also tend to stay at one party, making the job of tracking close contacts easier, as well as reducing the potential number of quarantined susceptible guests, *Sq*.

## Conclusion

The model presented in this paper illustrated the possible consequences of different regulations that could be imposed on the nightlife industry given a super spreader event. The results indicated the possible challenges, strengths and weaknesses of different strategies and should be a source for discussion when imposing regulations on local communities and constructing national guidelines and regulations.
